# Clinical and immunological characteristics of HIV/syphilis co-infected patients following long-term antiretroviral treatment

**DOI:** 10.3389/fpubh.2023.1327896

**Published:** 2024-01-15

**Authors:** Yuanni Wu, Lianfeng Lu, Xiaojing Song, Xiaosheng Liu, Yang Yang, Ling Chen, Jia Tang, Yang Han, Wei Lv, Wei Cao, Taisheng Li

**Affiliations:** ^1^Department of Infectious Diseases, Peking Union Medical College Hospital, Peking Union Medical College, and Chinese Academy of Medical Sciences, Beijing, China; ^2^Tsinghua-Peking Center for Life Sciences, Beijing, China; ^3^State Key Laboratory of Complex Severe and Rare Diseases, Peking Union Medical College Hospital, Chinese Academy of Medical Science and Peking Union Medical College, Beijing, China

**Keywords:** HIV, AIDS, syphilis, antiretroviral treatment, sexually transmitted disease, immune recovery

## Abstract

**Objective:**

This study aims to analyze the efficacy of anti-syphilis treatment and the impact of syphilis events on HIV virology and immunology in HIV/syphilis co-infected patients on long-term antiretroviral therapy (ART) and to investigate the incidence and factors of syphilis recurrence/re-infection/serofast state. The insights derived from this investigation can potentially guide strategies for preventing and managing syphilis and AIDS.

**Methods:**

A retrospective case–control study was conducted at the AIDS clinic of Peking Union Medical College Hospital from January 2003 to December 2022. The study involved 86 HIV/syphilis co-infected patients and 86 HIV mono-infected patients matched based on age, baseline CD4 + T cell counts, and viral load. We examined the clinical characteristics of HIV/syphilis co-infected patients, evaluated the efficacy of anti-syphilis treatment, and analyzed the dynamic changes in HIV virology and immunology. The Generalized Estimating Equations (GEE) model investigated the factors associated with HIV/syphilis co-infection and syphilis recurrence/reinfection/serofast state.

**Results:**

Syphilis serofast state was observed in 11.6% (10/86) of HIV/syphilis co-infected patients after treatment, and 33.7% (29/86) had syphilis recurrence or re-infection. The overall effectiveness of syphilis treatment stood at 76.8% (63/82). Notably, the effectiveness of syphilis treatment displayed a significant correlation with baseline syphilis titers exceeding 1:128 (*p* = 0.003). Over the 10-year follow-up period on ART, the HLA-DR + CD8+/CD8 + % levels in the HIV/syphilis co-infected group were markedly higher than those in the HIV mono-infected group (*p* < 0.05). However, no significant differences were observed between the two groups regarding HIV viral load, CD4+ T cell counts, CD8+ T cell counts, CD4/CD8 ratio, and CD38 + CD8+/CD8 + % (*p* > 0.05). GEE analysis model revealed that elevated HLA-DR + CD8+/CD8 + % levels were associated with HIV/syphilis co-infection (OR = 1.026, 95% CI = 1.007–1.046; *p* = 0.007) and syphilis recurrence/reinfection/serofast state (OR = 1.036, 95% CI = 1.008–1.065; *p* = 0.012).

**Conclusion:**

While HIV/syphilis co-infected patients typically receive adequate treatment, the incidence of syphilis recurrence and reinfection remain notably elevated. A heightened HLA-DR + CD8+/CD8+ % is a notable risk factor for HIV/syphilis co-infection and syphilis recurrence/reinfection/serofast state. Therefore, it is advisable to reinforce health education efforts and ensure regular follow-ups for people living with HIV undergoing ART to monitor syphilis infection or increased risk of syphilis infection.

## 1 Introduction

At present, both China and the global community confront the dual challenges posed by the syphilis and AIDS epidemics. According to the infectious disease surveillance data of the China Disease Control and Prevention Information System, as of the end of 2022, approximately 1.2 million living HIV/AIDS patients have been reported across the country, including 689,000 HIV-infected people and 534,000 AIDS patients (1). There were 480,020 syphilis patients in China in 2021, an increase of 15,585 cases compared to 2020 (2). The incidence of syphilis in HIV/AIDS patients is eight times higher than in the general population due to the similar transmission routes and infection groups (3). Literature shows that the rate of HIV and syphilis co-infection has been increasing over the last decade (4). In Ontario, Canada, the co-infection prevalence showed a steady increase from 1.8 per 100 person-years in 2006 to 4.3 per 100 person-years in 2010 (5). Similarly, in Shenzhen, China, this prevalence surged from 13.13% in 2013 to 20.9% in 2015 (6, 7). Preventing and controlling co-infection with HIV and syphilis is still a challenge.

The reciprocal relationship between syphilis and HIV is often described as “epidemiological synergy” (8). HIV/syphilis co-infection had a negative impact on CD4 + T cell counts and HIV-RNA levels (9–14). Compared with pre-syphilis levels, CD4 + T counts decreased during syphilis range from 28 to 106 cells/uL (9, 11–13), while viral load increased on average from 0.22 to 0.26 RNA log 10 copies/ml (11, 12). However, the impact of syphilis infection on the immunological and virological aspects of people living with HIV (PLWH) undergoing long-term antiretroviral therapy (ART) remains uncertain. Furthermore, the outcomes of early syphilis treatment in PLWH exhibit inconsistencies. While some studies have reported higher failure rates in PLWH with syphilis following standard treatment (15, 16), others have not (17, 18). These conflicting findings can be attributed to limitations in the published research, including small sample sizes, inadequate stratification for different syphilis clinical stages, varying definitions of serological failure, high rates of patient loss to follow-up, and the inclusion of patients with low non-treponemal titers.

The rising incidence of syphilis recurrence and reinfection constitutes a notable aspect of the syphilis epidemic (19–21). Research has identified MSM (men who have sex with men), transgender individuals, individuals aged 20–39, and engaging in condomless sex as risk factors for recurrent syphilis in HIV-infected individuals (22). Therefore, this study sought to investigate the following: (1) the effectiveness of anti-syphilis treatment in HIV/syphilis coinfected individuals; (2) alterations in virological parameters, T cell subsets, and immune activation in PLWH after initiating ART in the context of syphilis; and (3) the prevalence and associated risk factors of syphilis recurrence and reinfection among PLWH undergoing long-term ART.

## 2 Patients and methods

### 2.1 Study design

A case–control study was conducted at Peking Union Medical College Hospital (PUMCH). Eighty-six HIV/syphilis co-infected patients who received antiretroviral treatment at the PUMCH AIDS Clinic from 2003 to December 31, 2022, were selected as the case group. Moreover, 86 HIV-infected individuals without syphilis matched with age, gender, baseline HIV viral load, and baseline CD4 + T cell counts were selected as the control group. The control group was recruited in the same Clinic and in the same period of the cases. The inclusion criteria were as follows: (1) Patients who received a diagnosis of HIV infection by the Chinese Guidelines for the Diagnosis and Treatment of HIV/AIDS (2021 Edition) (23); (2) Patients who underwent baseline assessments for HIV viral load and T cell subsets, with positive results on both the *Treponema Pallidum* Particle Assay (TPPA) and rapid plasma regain (RPR) tests in the case group and negative in the control group; (3) Patients who had at least one measurement of HIV viral load, T cell subset, and syphilis RPR titer during the follow-up period.

The exclusion criteria for the case and control group included: (1) Individuals under the age of 18; (2) Insufficient data regarding baseline and follow-up measurements of HIV load, CD4+ T cell counts, and syphilis antibodies; (3) Pregnant women.

The studies involving humans were approved by the Peking Union Medical College Hospital Ethics Review Committee (ID: 1-23PJ1189). The studies were conducted in accordance with the local legislation and institutional requirements. The participants provided their written informed consent to participate in this study.

### 2.2 Methods

Demographic and epidemiological data of AIDS outpatients were gathered through the electronic medical record system at PUMCH. This information included age, gender, ethnicity, mode of HIV transmission, date of HIV diagnosis, ART commencement, and more. The HIV-RNA plasma viral load was assessed utilizing the COBAS Ampliprep/TaqMan 48 RT-PCR real-time test (Roche, CA, United States), with the lower limit of detection set at 20 copies/mL for HIV-1 viral load. Flow cytometer (LSR Fortessa, BD Biosciences) was used to obtain lymphocyte subsets, and FlowJo software (Tree Star, Ashland, OR United States) was utilized for data analysis. To conduct TPPA, the reagents were acquired from the British OMEGA Company. To conduct RPR, the reagents were obtained from Urumqi High-tech Industrial Development Zone Xindi Company. Patients had regular clinic visits at 4, 12, 24, 36, and 48 weeks after ART initiation. Subsequently, patients visited our clinic at least once every 3 months to monitor lymphocyte subsets and HIV-1 viral load.

### 2.3 Definition of syphilis infection

The diagnosis and staging of syphilis adhere to the classification provided by the Centers for Disease Control and Prevention (24). Syphilis recurrence or reinfection is determined by a ≥ 4-fold increase in RPR titer after initial syphilis treatment, as observed during later follow-up (25). A syphilis serofast state characterizes patients who, after receiving regular anti-syphilitic treatment, maintain a persistently low titer level (≤1: 4) in peripheral blood RPR tests, which may even remain positive for life.

### 2.4 Definition of group

The definition of each group is as follows:

**Table:** 

Group	Definition
Co-infection group	HIV and syphilis coinfected patients
Mono-infection group	Patients with only HIV infection but no syphilis infection
A group (syphilis recurrence/reinfection/serofast state group)	Patients with syphilis recurrence/reinfection/serofast state after treatment
B group	Patients without syphilis recurrence/reinfection/serofast state after treatment

### 2.5 Statistical analysis

Statistical analyses were conducted using SPSS (version 22.0, SPSS Inc., Chicago, IL, United States), and GraphPad Prism 8.0 (GraphPad Software, Inc., La Jolla, CA, United States) was utilized for data visualization. Descriptive statistics are reported as the mean with standard deviation (SD) or median with interquartile range (IQR). The Mann–Whitney U test was employed for comparing continuous variables between two groups. We applied Generalized Estimating Equations (GEE) to assess the risk factors associated with HIV/syphilis co-infection and syphilis recurrence/reinfection. Statistical significance was defined as a value of p less than 0.05.

## 3 Results

### 3.1 Characteristics of the patients included in the study

The average age of the HIV/syphilis co-infection and HIV mono-infection groups (abbreviated as case and control group) was 34.9 ± 10.0 and 35.0 ± 12.0 years, respectively (*p* = 0.615). Both groups consisted entirely of male participants, with sexual contact as the primary transmission route (91.9% in the case group and 86% in the control group). The median ART duration was 8.8 (6.1, 10.8) years for the case group and 9.9 (8.0, 11.5) years for the control group. For the case group, the baseline HIV viral load was4.71 ± 0.66 log10 copies/mL, while for the control group, it was 4.79 ± 0.62 log10 copies/mL. The median baseline CD4 + T counts for the case group were 255 (166,357) cells/uL and 256 (175,373) cells/uL for the control group. There were no significant differences in age, gender, transmission route, ART regimen, baseline HIV viral load, baseline CD4 + T cell counts, baseline CD8 + T cell counts, baseline CD4/CD8 ratio, baseline HLA-DR + CD8+/CD8 + %, and baseline CD38 + CD8+/CD8 + % between the two groups (Table 1).

**Table 1:** Demographic characteristics and clinical detection indicators of HIV/syphilis co-infection and HIV mono-infection groups.

Characteristics	HIV/syphilis co-infection			HIV mono-infection *N* = 86	*p* value
	Total *N* = 86	Serofast state *N* = 10	Recurrence/reinfection *N* = 29		
Age, mean years	34.9 ± 10.0	41.7 ± 12.3	33.7 ± 8.0	35.0 ± 12.0	0.615
Route of transmission, n(%)					0.127
Sexual	79 (91.9%)	10 (100%)	28 (96.6%)	76 (86.0%)	
Blood	0 (0%)	0 (0%)	0 (0%)	4 (7.0%)	
Other	7 (8.1%)	0 (0%)	1 (3.4%)	6 (7.0%)	
Baseline HIV Viral load	4.71 ± 0.66	4.22 ± 0.84	4.66 ± 0.60	4.79 ± 0.62	0.544
(log10 copies/mL)					
Baseline CD4 + T count(cells/uL)	255 (166,357)	250 (217,419)	255 (191,356)	256 (175,373)	0.825
Baseline CD8 + T count(cells/uL)	969 (689,1,278)	770 (621,1,146)	873 (677,1,241)	875 (626,1,273)	0.535
CD8 + CD38+/CD8+ T cell at Baseline (%)	79.9 (71.0,87.8)	84.5 (68.1,87.6)	78.8 (62.5,86.5)	79.6 (68.5,86.4)	0.638
CD8 + HLA-DR+/CD8+ T cell at Baseline (%)	62.3 (52.7,76.4)	61.5 (58.5,75.9)	62.3 (56.4,76.7)	63.8 (51.9,70.7)	0.539
Baseline CD4/CD8 ratio	0.26 (0.17,0.38)	0.37 (0.26,0.53)	0.30 (0.21,0.44)	0.27 (0.17,0.48)	0.65
ART regimen [n(%)]					0.201
2NRTIs + NNRTI	62 (72.1%)	9 (90.0%)	24 (82.8%)	64 (74.4%)	
2NRTIs + PI	5 (5.8%)	0 (0%)	0 (0%)	7 (8.1%)	
2NRTIs + INSTI	16 (18.6%)	1 (10.0%)	4 (13.8%)	8 (9.4%)	
Other	3 (3.5%)	0 (0%)	1 (3.4%)	7 (8.1%)	
Years since ART (years)	8.8 (6.1,10.8)	9.0 (7.4,9.6)	10.2 (7.3,11.2)	9.9 (8.0, 11.5)	0.003
Highest RPR titer	512	64	256	–	–
Treatment regimen[n(%)]				–	–
Benzylpenicillin	51 (81.0%)	5 (55.6%)	22 (88%)		
Tetracyclines	10 (15.9%)	3 (33.3%)	3 (12%)		
Other	2 (3.1%)	1 (11.1%)	0 (0%)		

NRTIs, nucleoside reverse transcriptase inhibitors; NNRTI, Non-nucleoside reverse transcriptase inhibitor; INSTI, integrase strand transfer inhibitor; PI, protease inhibitor; IQR, interquartile range. *P* value, HIV/syphilis co-infection group vs. HIV mono-infection group.

There were 82 treated syphilis patients and four untreated patients in the case group. The highest RPR titer observed in patients was 1:512. Among 63 documented patients, 51 (81.0%) received primary treatment with Benzylpenicillin, while treatment drugs were missing for 19. After treatment, 10 patients (11.6%) remained in a serofast state, and 29 patients (33.7%) experienced recurrence or reinfection, as depicted in Table 1.

### 3.2 Therapeutic effect of anti-syphilis treatment in HIV/AIDS patients

Regarding anti-syphilis treatment, benzylpenicillin was given to 51 patients, minocycline to 5 patients, doxycycline to 4 patients, intensive treatment combining benzylpenicillin and minocycline to 1 patient, streptomycin to 1 patient, and tetracycline to 1 patient. The overall effectiveness of the treatment was 76.8% (63/82). In subgroup analysis, no significant correlation emerged between the efficacy of anti-syphilis treatment and age, HIV viral load, baseline CD4+ T cell counts, baseline CD8+ T cell counts, or baseline CD4/CD8 ratio in HIV/AIDS patients; the differences were not statistically significant. However, the efficacy of anti-syphilis treatment in patients with a baseline syphilis titer higher than 1:128 was only 45.5%, significantly lower than that in patients with a baseline syphilis titer lower than 1:128 (*p* = 0.003), as illustrated in Table 2.

**Table 2:** Treatment efficiency of subgroups of patients with HIV co-infected syphilis.

Subgroups	Case	Yes	No	Efficiency	$x^{2}$	*p* value
Age(years)						
19 ~ 35	52	44	8	84.60%	2.185	0.344
36 ~ 50	25	20	5	80.00%		
51~	5	3	2	60.00%		
Baseline CD4 + T cell counts (cells/uL)					0.583	0.542
<200	26	20	6	76.90%		
≥200	56	47	9	83.90%		
HIV Viral load (log 10 copies/mL)					0.147	0.757
≥5	24	19	5	82.80%		
<5	58	48	10	79.20%		
Highest RPR titer					12.361	0.003
≥128	11	5	6	45.50%		
<128	70	62	8	88.60%		
Baseline CD8 + T cell counts (cells/uL)					1.489	0.478
<500	10	7	3	70.00%		
501 ~ 1,000	37	30	7	81.10%		
1,001~	35	30	5	85.70%		
Baseline CD4/CD8 ratio					0.001	1
<0.3	44	36	8	81.80%		
≥0.3	38	31	7	81.60%		

### 3.3 HIV-related virological and immunological dynamics in two groups

**Figure 1:**
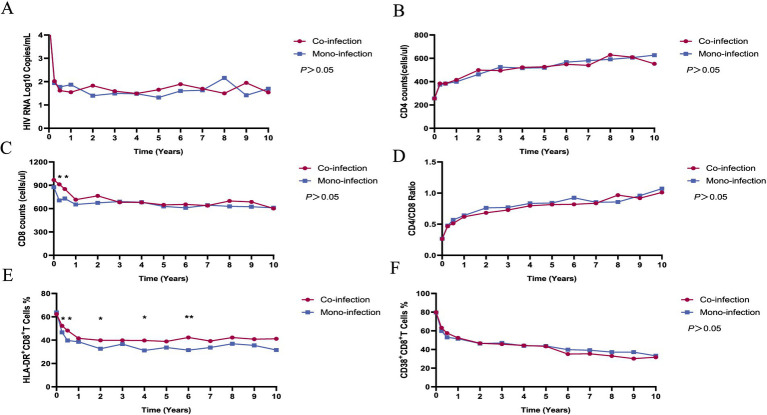
The dynamics of virological and immunological parameters in HIV/syphilis co-infection and HIV mono-infection groups after long-term ART. Red and blue lines indicate the fluctuations of HIV/syphilis co-infection and HIV mono-infection groups in the different visits. The plasma HIV-1 RNA levels **(A)**, CD4+ T cell counts **(B)**, CD4+/CD8+ ratio **(D)**, and CD38 + CD8+/CD8 + % **(F)** did not differ significantly between the two groups. The CD8+ T cell counts **(C)** were higher in the HIV/syphilis co-infection group compared to the HIV mono-infection group at three and 6 months after initiating ART (*p* = 0.01, 0.03, respectively). The levels of HLA-DR + CD8+/CD8 + % **(E)** in the HIV/syphilis co-infection group were substantially elevated compared to those in the HIV mono-infection group at 3, 6 months, 2, 4, and 6 years, with *p* values of 0.018, 0.014, 0.044, 0.027, and 0.004, respectively.

The virological and immunological profiles of participants in both the case and control groups are depicted in Figure 1. In line with prior research, a rapid reduction in HIV RNA levels and a concurrent rise in CD4+ T cell counts were observed within the first year following the initiation of ART in both groups. Decreased CD8+ T cell counts, HLA-DR + CD8+/CD8 + % ratios, and CD38 + CD8+/CD8 + % levels often accompany these changes.

Throughout the extended 10-year follow-up period, no statistically significant distinctions emerged between the two groups in terms of HIV RNA levels, CD4+ T cell counts, CD4/CD8 ratios, or CD38 + CD8+/CD8 + % values (*p* > 0.05). However, at three and 6 months after initiating ART, the CD8+ T cell counts were higher in the HIV/syphilis co-infection group compared to the HIV mono-infection group (*p* = 0.01, 0.03, respectively; Figures 1B,C). Notably, the levels of HLA-DR + CD8+/CD8 + % in the HIV/syphilis co-infection group were substantially elevated compared to those in the HIV mono-infection group at 3, 6 months, 2, 4, and 6 years, with *p* values of 0.018, 0.014, 0.044, 0.027, and 0.004, respectively (Figure 1E). Additionally, further analysis employing a GEE model indicated that HLA-DR + CD8+/CD8 + % ratios were significantly associated with co-infection patients [OR = 1.026, 95% confidence interval (CI) = 1.007–1.046; *p* = 0.007; Table 3].

**Table 3:** GEE analysis of influencing factors of uncontrolled syphilis.

Variables	Syphilis recurrence/reinfection/serofast state para. Estimates QIC: 1102.289; QICC: 1027.401		HIV/syphilis co-infection para. Estimates QIC: 2242.441; QICC: 2166.140	
	Odds ratio (95% CI)	*P* value	Odds ratio (95% CI)	*P* value
(Intercept)	0.601 (0.041 ~ 8.918)	0.712	0.594 (0.111 ~ 3.175)	0.542
Age (year)	0.980 (0.929 ~ 1.033)	0.443	0.991 (0.960 ~ 1.022)	0.547
HIV RNA (log10 copies/ml)	0.893 (0.757 ~ 1.053)	0.179	0.907 (0.806 ~ 1.022)	0.109
CD4 count (cells/μL)	0.999 (0.996 ~ 1.002)	0.468	1.001 (1.000 ~ 1.003)	0.062
CD8 count (cells/μL)	1.000 (0.999 ~ 1.001)	0.967	1.000 (0.999 ~ 1.000)	0.457
HLA-DR+/CD8 + (%)	1.036 (1.008 ~ 1.065)	0.012	1.026 (1.007 ~ 1.046)	0.007
CD38+/CD8 + (%)	0.989 (0.974 ~ 1.005)	0.192	0.997 (0.986 ~ 1.009)	0.667
CD4/CD8 ratio	2.387 (0.444 ~ 12.832)	0.311	0.865 (0.757 ~ 0.998)	0.082

Para., parameter; QIC, Quasi-Likelihood under the Independent Model Criterion; QICC, Adjusted Quasi-Likelihood under Independent Model Criterion.

### 3.4 Analyzing the factors of syphilis recurrence, reinfection, and serofast state

**Figure 2:**
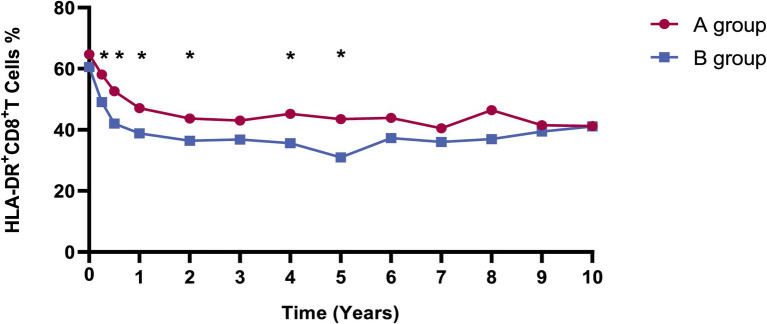
The dynamics of HLA-DR + CD8+/CD8 + % in A and B groups. Red and blue lines indicate the fluctuations of A and B groups in the different visits. The levels of HLA-DR + CD8+/CD8 + % in the A group were substantially elevated compared to those in the B group at 3, 6 months, 1, 2, 4, and 5 years, with *p* values of 0.035, 0.018, 0.044, 0.045, 0.011, and 0.012, respectively. Group A: Patients with syphilis recurrence/reinfection/serofast. Group B: Patients without syphilis recurrence/reinfection/serofast.

Considering the relatively higher incidence of syphilis recurrence/reinfection in our study and to explore further the impact of syphilis treatment on immune recovery and activation in HIV patients, we compared immunological markers in patients with recurrent/reinfection or serofast state (group A) and those remaining who without recurrent/reinfection or serofast state (group B). The analyses revealed no significant differences between the two groups in CD4+ T cell counts, CD8+ T cell counts, and CD8 + CD38 + % over up to 10 years of follow-up (*p* > 0.05), as depicted in Supplementary Figure 1. Notably, the level of HLA-DR + CD8+/CD8 + % in A group was substantially higher than that in B group, as illustrated in Figure 2.

We employed GEE models with repeated laboratory measurements to delve deeper into the factors impacting syphilis recurrence/reinfection/serofast state during long-term ART (Table 3). The findings indicated a positive correlation between the syphilis recurrence/reinfection/serofast state with HLA-DR + CD8+/CD8 + % (OR = 1.036, 95%CI = 1.008–1.065; *p* = 0.012). However, no correlations were observed with age, CD4 + T cell counts, CD8 + T cell counts, or CD38 + CD8+/CD8 + %.

## 4 Discussion

HIV and syphilis share similar transmission routes and are prevalent among high-risk populations. Consequently, they can mutually exacerbate disease progression, leading to an escalating incidence of new HIV co-infections with syphilis in recent years (26). Many reports introduced prevalence or traditional risk factors for HIV/syphilis coinfection (7, 27, 28). Our study found that baseline RPR titers exceeding 1:128 indicate a higher risk of syphilis treatment failure, consistent with prior studies. However, we comprehensively examined the immunological and virological change during 10 years of ART. We innovatively employed the GEE model to analyze the correlation between HIV/syphilis co-infection and syphilis recurrence/reinfection/serofast state and T cell activation. We found that higher HLA-DR + CD8+/CD8% during long-term ART is associated with syphilis co-infection and the recurrence/reinfection/serofast state of syphilis following treatment.

In prior retrospective studies conducted during the ART era, PLWH coinfected with syphilis displayed serological response rates to Benzylpenicillin ranging from 70.4 to 91.8% (15–18). Our study findings indicate that syphilis treatment is generally effective in individuals concurrently diagnosed with HIV and syphilis, with an overall serologic remission rate of 76.8%. This is consistent with the findings of Yang et al. (with an overall serological remission rate of 70.9%) (29). Notably, instances of syphilis treatment failure were associated with baseline RPR titers exceeding 1:128. These insights serve as valuable references for managing syphilis infection in HIV-infected individuals.

The conclusions of existing studies regarding whether syphilis infection affects CD4+ T cell recovery and immune activation in HIV patients receiving ART remain inconclusive. While some studies have indicated that syphilis infection leads to a decline in CD4+ T cell counts in HIV-infected individuals (9, 11) and that HIV/syphilis co-infection hampers immune recovery and antiretroviral effectiveness (30), others, such as Wang et al. (31) have reported that patients at different stages of disease progression experience an increase in CD4 + T cell counts of more than 200 cells/μL after treatment. Consistent with Wang et al.’s findings (31), our study demonstrates that HIV/syphilis co-infected patients, including cases of uncontrolled syphilis, do not experience adverse effects on HIV viral load suppression, the recovery of CD4+ T and CD8+ T cell counts, or the levels of CD8+ CD38+/CD8 + % during extended ART. Notably, throughout a 10-year ART follow-up, we observed an intriguing trend: HLA-DR + CD8+/CD8 + % levels were consistently higher in HIV/syphilis co-infected patients than those with HIV mono-infection. This phenomenon may be attributed to the potential of syphilis infection to enhance the activation of host immune cells by modifying cytokine secretion and upregulating transcription factors (32).

Earlier research has demonstrated that among the MSM population, the incidence of syphilis recurrence/reinfection in HIV/AIDS patients is substantially higher than in non-HIV MSM individuals (29% vs. 16%) (28). Similarly, Courjon et al.’s study reported an incidence of 28% for HIV and syphilis recurrence/reinfection in the MSM population after a 24-month follow-up period (33). In our study, we observed an incidence of syphilis recurrence/reinfection at 33.7%, slightly higher than 30% of other reports. This may be attributed to our extended follow-up duration and a relatively modest sample size.

Several traditional factors contribute to syphilis recurrence/reinfection within the HIV/AIDS population, including a high RPR titer (>1:64) at baseline (34), delayed initiation of ART, younger age, and engagement in unprotected sexual behavior (22). In our research, adjusting for traditional risk factors, a heightened HLA-DR + CD8+/CD8 + % level emerges as a notable risk factor for syphilis recurrence, reinfection, or serofast. This marks the inaugural evidence linking T-cell activation to syphilis relapse or recurrence in individuals co-infected with HIV and syphilis.

Nevertheless, this study does possess certain limitations. Firstly, given its real-world observational nature, variations in patient follow-up durations and occasional dropouts may introduce data deviation. Secondly, the follow-up intervals, primarily 3 to 6 months, occasionally relied on patient-reported incidents for assessing syphilis recurrence and relapse, possibly introducing subjectivity. Thirdly, the small sample size and predominantly males limit the conclusions’ generalizability to the broader population. Lastly, because our study solely collected routine laboratory parameters and had sample size constraints, we could not delve into comprehensive discussions on more risk factors like patients’ drug treatment adherence and lifestyle and underlying mechanisms.

In conclusion, this analysis was based on an HIV/ syphilis co-infection cohort with the most extended follow-up period, featuring the most comprehensive and regular measurements. In HIV/ syphilis co-infected individuals on long-term ART, syphilis treatment failure was associated with high baseline RPR titers. In contrast, syphilis events did not adversely affect HIV viral suppression and immune reconstitution compared to mono-HIV-infected patients. In addition, a higher frequency of syphilis reinfection was observed during the 10-year follow-up period, and the level of HLA-DR + CD8+/CD8 + % was positively correlated with HIV/syphilis co-infection and recurrence/reinfection/serofast state of syphilis. Overall, HIV/AIDS patients co-infected with syphilis should promptly receive standardized syphilis treatment alongside ART. Regular follow-up assessments, including CD4 + T cells and immune activation, ensure robust immune system function recovery and enhance overall quality of life.

## Data availability statement

The raw data supporting the conclusions of this article will be made available by the authors, without undue reservation.

## Ethics statement

The studies involving humans were approved by The Ethics Committee of Peking Union Medical College Hospital. The studies were conducted in accordance with the local legislation and institutional requirements. The participants provided their written informed consent to participate in this study.

## Author contributions

YW: Data acquisition, Data curation, Methodology, Writing – original draft, Writing – review & editing. LL: Data acquisition, Data curation, Methodology, Writing – original draft, Writing – review & editing. XS: Data acquisition, Data curation, Writing – review & editing. XL: Writing – review & editing. YY: Writing – review & editing. LC: Writing – review & editing. JT: Writing – review & editing. YH: Methodology, Writing – review & editing. WL: Data acquisition, Data curation, Writing – review & editing. WC: Data acquisition, Writing – review & editing. TL: Funding acquisition, Supervision, Writing – review & editing.
