# Supplementary material for: Clinical and immunological characteristics of HIV/syphilis co-infected patients following long-term antiretroviral treatment
Source: Front Public Health. 2024 Jan 15;11:1327896. doi: 10.3389/fpubh.2023.1327896 (PMC10823526; doi:10.3389/fpubh.2023.1327896)
Supplement: Supplementary file 1 [file Image_1.pdf]

Supplementary Material

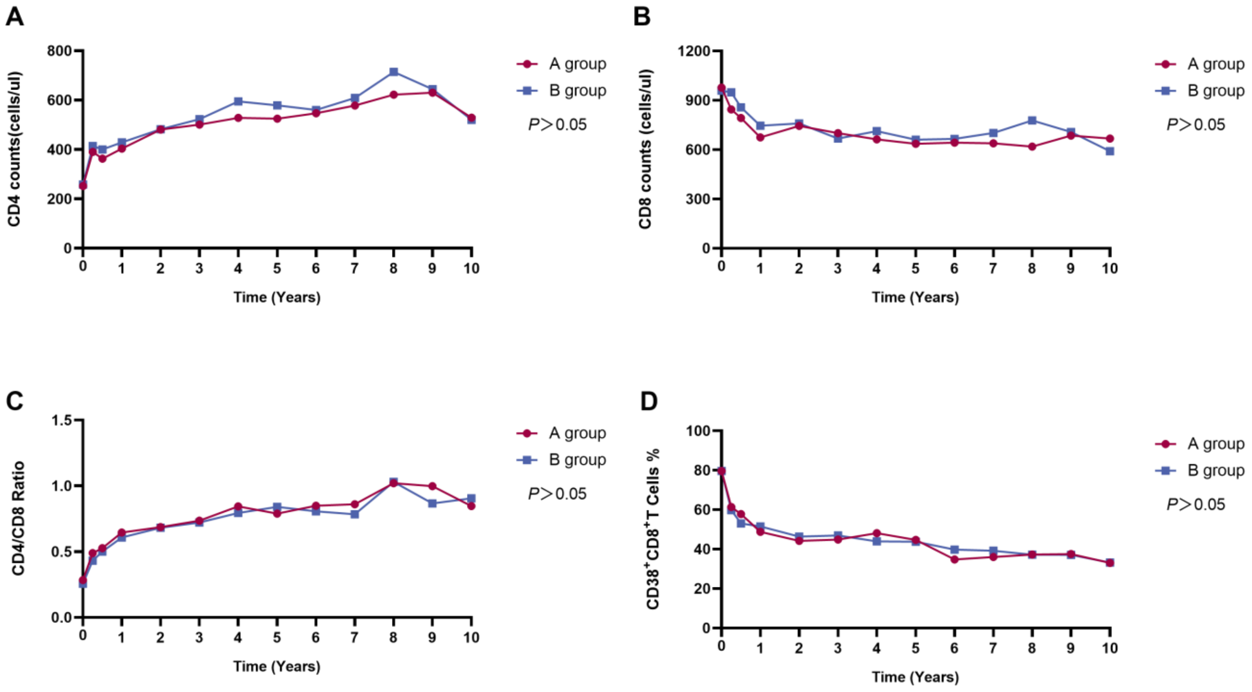

Supplementary Figure 1. The dynamics of immunological parameters in A and B groups. Red and blue lines indicate the fluctuations of A and B groups in the different visits. The CD4+ T cell counts (A), CD8+ T cell counts(B), CD4+/CD8+ ratio(C), and CD38+CD8+/CD8+% (D) did not differ significantly between the two groups($P > 0.05$).Group A: Patients with syphilis recurrence/reinfection/serofast state. Group B: Patients without syphilis recurrence/reinfection/serofast state.
